# Mapping the global research landscape and innovations on elderly glioma: a bibliometric analysis

**DOI:** 10.3389/fonc.2026.1769459

**Published:** 2026-03-27

**Authors:** Shaochun Guo, Wenbo Zhao, Jinghui Liu, Na Wang, Peigang Ji, Liang Wang, Yuan Wang

**Affiliations:** 1 Department of Neurosurgery, Tangdu Hospital, Airforce Medical University, Xi’an, China; 2 Department of Neurosurgery, Second Hospital of Shanxi Medical University, Taiyuan, China

**Keywords:** CiteSpace, elderly, glioma, scientometrics, VOSviewer

## Abstract

**Objective:**

Glioma demonstrates age-specific molecular profiles and heightened aggressiveness in older adults, yet research remains predominantly focused on younger populations. Amid global aging and rising incidence, tailored therapeutic strategies for elderly patients are urgently needed. This study provides a comprehensive bibliometric analysis of scientific literature on glioma in the elderly, aiming to map research output, identify knowledge gaps, and highlight interdisciplinary innovations.

**Methods:**

We conducted a systematic search in the Web of Science Core Collection, finding 1,299 relevant publications, and reviewed randomized controlled trials from PubMed to assess clinical progress. We used CiteSpace and VOSviewer to analyze temporal trends, contributions by countries and institutions, collaboration networks, journal impact, co-citations, keyword clusters, and emerging research frontiers.

**Results:**

Annual publications increased 4.3-fold from 2001 to 2025. The U.S. (32.8%), Italy (12.4%), and Germany (11.0%) were the leading contributors. Research evolution progressed through three phases: histopathological classification (2001–2013), therapy standardization with radiotherapy/temozolomide (2014–2018), and molecular stratification focusing on MGMT methylation (2019–2025). Key institutions included the University of Zurich and Mayo Clinic. Keyword clustering highlighted aging-related priorities such as surgical management, geriatric assessment, and molecular phenotypes.

**Conclusion:**

Significant gaps persist in elderly glioma research, particularly regarding age-related comorbidities and molecular heterogeneity. While clinical trials have established treatment frameworks, future studies should integrate geriatric assessments, tumor microenvironment dynamics, and cross-disciplinary approaches. Dominance by Western institutions underscores opportunities for global collaboration, especially with rapidly aging nations.

## 1 Introduction

Gliomas, as classified by the World Health Organization (WHO) into grades I to IV according to their histological characteristics and malignancy levels, originate from neuroglial cells and constitute the most prevalent form of primary central nervous system (CNS) tumors. These tumors are distinguished by their infiltrative growth patterns, disruption of the blood-brain barrier (BBB), and abnormal angiogenesis, which present substantial challenges in neurosurgical practice due to their high morbidity and mortality rates (1, 2).

The global trend of population aging has contributed to a rise in the incidence of gliomas among the elderly population (3). Research indicated that the incidence rate of gliomas in individuals aged 65 and older ranges from 5 to 8 per 100,000 persons, with both the incidence and malignancy of these tumors increasing with advancing age (4). Molecular biological investigations have identified distinct differences between gliomas in elderly patients and those in younger cohorts. Notably, gliomas in the elderly are more frequently associated with epidermal growth factor receptor (EGFR) amplification (5) and aberrant activation of the phosphatidylinositol 3-kinase (PI3K)/protein kinase B (AKT) signaling pathway (6, 7). For MGMT promoter methylation, its role in elderly cohorts is influenced by a range of factors, including the extent of methylation, co-existing molecular alterations, and treatment regimens (8, 9). These molecular characteristics have significant implications for tumor diagnosis, therapeutic strategies, and prognosis.

Elderly patients often have comorbidities (e.g., cardiovascular disease, frailty) that limit treatment tolerance (10). Historical RCTs excluded patients >70 years or with KPS <70, creating evidence gaps for the majority of elderly patients; median age in landmark Stupp trial was 56 years (3). Therefore, research on gliomas in the elderly remains relatively underexplored. In addition, elderly glioma care involves complex geriatric assessment, polypharmacy management, and palliative integration distinct from curative-focused younger adult protocols. Bibliometric analysis specifically targeting this population addresses a systematically underrepresented research domain. In light of this gap, conducting a bibliometric analysis of literature pertaining to gliomas in the elderly is of importance. This study seeks to systematically examine the global literature output, research hotspots, and emerging trends in the field of gliomas in the elderly. The objective is to provide a comprehensive overview of the current research landscape, identify existing deficiencies, and furnish valuable insights for researchers and clinicians. Such an analysis will contribute to the development of clinical guidelines and research policies specifically tailored to the needs of elderly glioma patients.

## 2 Materials and methods

### 2.1 Search strategies and dataset establishment

On 20 July 2025, a comprehensive literature search was performed using the Web of Science Core Collection (WoSCC) database to identify pertinent publications related to glioma in the elderly, encompassing studies published from 1 January 2001 to 30 June 2025. The WoSCC is esteemed for its extensive and thorough coverage of high-quality scientific literature and is acknowledged as the most widely accepted and utilized database for bibliometric analysis.

The WoScc serves as a multidisciplinary database, whereas PubMed, managed by the U.S. National Library of Medicine, is specifically focused on clinical research. For our federated analysis, we also utilized PubMed due to its proficiency in aggregating clinical research data and facilitating access to clinical trials. A systematic search conducted in PubMed, employing the Randomized Controlled Trial filter, identified 304 PubMed-indexed RCT records related to glioma research in the elderly, thereby underscoring the advancements in clinical research within this field.

**Figure 1:**
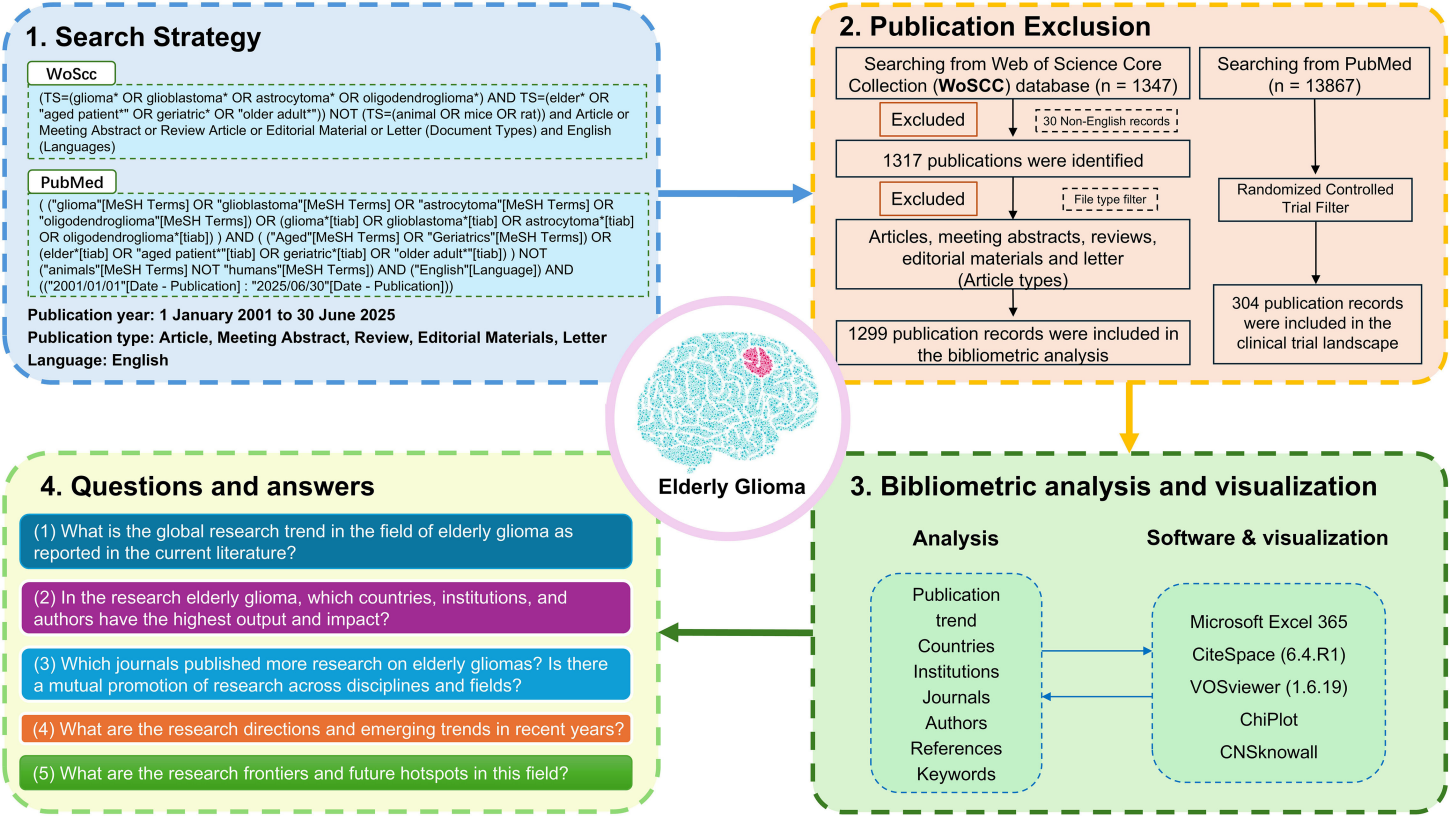
Flow diagram and overview of the current study.

The search strategy was meticulously designed to maximize specificity and relevance to the research topic, employing the following criteria: (1) Search terms: The topic (TS) included the keywords “glioma* OR glioblastoma* OR astrocytoma* OR oligodendroglioma*” in conjunction with “elder* OR “aged patient*” OR geriatric* OR “older adult*”. (2) Document types: To ensure academic rigor and relevance, the search was restricted to meeting abstracts, articles, review, editorial material and letter. (3) Language: Only English-language manuscripts were considered for inclusion in this analysis. The search was performed independently by SCG and WBZ with 100% agreement on final inclusion. Specific exclusions: To prevent contamination from non-parenchymal CNS tumors, we explicitly excluded terms including ‘orbital,’ ‘meningioma*,’ ‘schwannoma*,’ and ‘pituitary*’ through NOT statements in preliminary search iterations (**Supplementary Table 1**). Animal studies excluded via: NOT TS=(‘animal*’ OR ‘mice’ OR ‘rat*’ OR ‘*in vivo*’ OR ‘cell line’). The detailed methodology of this search was illustrated in **Figure 1** and **Supplementary Table 1**. The raw data supporting the conclusions of this article could be found in **Supplementary File**.

Age threshold definition: Given the absence of standardized age criteria for ‘elderly’ glioma patients across the 2001–2025 literature, we employed an inclusive search strategy capturing all studies using age-related descriptors (elderly, aged, geriatric, older adult) without imposing a fixed numerical cutoff. This approach ensures comprehensive coverage of the field’s evolution, as age thresholds have varied historically (≥60 to ≥75 years) and remain study-dependent in clinical practice. *Post-hoc* analysis of included randomized controlled trials confirmed that most of studies used thresholds between 60–70 years, with ≥65 years being predominant in publications after 2012.

### 2.2 Bibliometric analysis and visualization

CiteSpace (6.4.R1 advanced), VOSviewer (version 1.6.19), Pajek (Version 6.01) and SClMago Graphica (Version 1.0.51) were employed as robust software tools to generate bibliometric maps from network data. In our analysis, we utilized network visualization and overlay visualization techniques to construct networks that encompass countries, institutions, journals, authors, references, and keywords, in addition to conducting burst analysis on journals, references, and keywords.

In this study, CiteSpace was utilized with time slices ranging from January 2001 to June 2025, with each slice representing a single calendar year. VOSviewer facilitated bibliometric analyses, encompassing evaluations of institutions, authors, journals, keywords, and references.

All network visualizations were generated with documented parameter settings to ensure reproducibility. CiteSpace parameters (all analyses): time slicing: 2001–2025, 1 year per slice; selection criteria: top 50 most-cited references per slice; top 20% high-frequency keywords; network pruning: Pathfinder algorithm; cluster labeling: Log-likelihood ratio (LLR), top N = 3; burst detection: Kleinberg’s algorithm, minimum duration=2 years, γ=1.0. VOSviewer parameters: association strength normalization; resolution: γ=0.8 (keywords), γ=1.0 (countries/institutions); minimum cluster size: 5 nodes; attraction/repulsion: 2/–1.

The analysis results of bar graph, bubble plot, Cleveland plot and radar chart were generated using the CNSknowall platform (https://cnsknowall.com) and ChiPlot (https://www.chiplot.online), which were comprehensive web services for data analysis and visualization.

### 2.3 Methodological transparency measures

To enhance reproducibility, we implemented the following: (1) comprehensive documentation of all search parameters and dates; (2) utilization of multiple software packages (CiteSpace, VOSviewer) to achieve convergent verification of cluster structures; (3) detailed reporting of algorithm-specific parameters and validation metrics; (4) execution of sensitivity analyses concerning database selection, document type, and age threshold; (5) provision of raw data and search logs in the **Supplementary Materials**. These measures are designed to address analytical consistency and transparency, rather than ‘validity’ in the positivist sense. We explicitly acknowledge that bibliometric findings reflect patterns in publication and citation behavior, rather than objective truths about scientific knowledge.

In conclusion, the implementation of this rigorous validation framework substantially enhances the credibility of the research findings and ensures their utility as a dependable resource for scholars, practitioners, and policymakers focused on elderly gliomas.

## 3 Results

### 3.1 Analysis of publication quantity and time distribution

**Figure 2:**
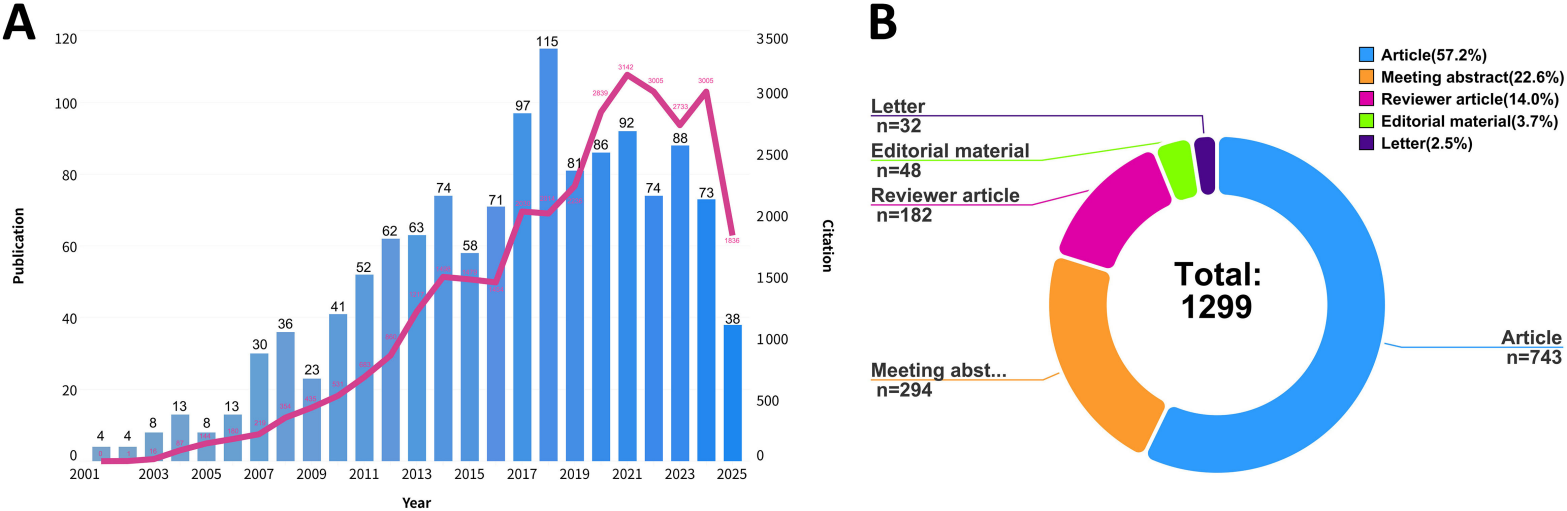
**(A)** Annual publication numbers from 1 January 2001 to 30 June 2025. **(B)** Distribution of document types by category.

Conducting a quantitative analysis of publications within a specific field served as an essential method for identifying significant trends and areas of focus. In this study, we examined a total of 1,299 publications, which included 743 articles (57.2%), 294 meeting abstracts (22.6%), 182 reviews (14.0%), 48 editorial material (3.7%) and 32 letters (2.5%) (**Figure 2**). The temporal distribution of research publications in elderly gliomas revealed a fluctuating yet generally upward trajectory. The annual publication increased 4.3-fold from 2001 to 2025. The first peak was observed in 2008 with 36 publications. After a decrease in 2015, the publication count peaked again in 2018 with 115 publications.

**Figure 3:**
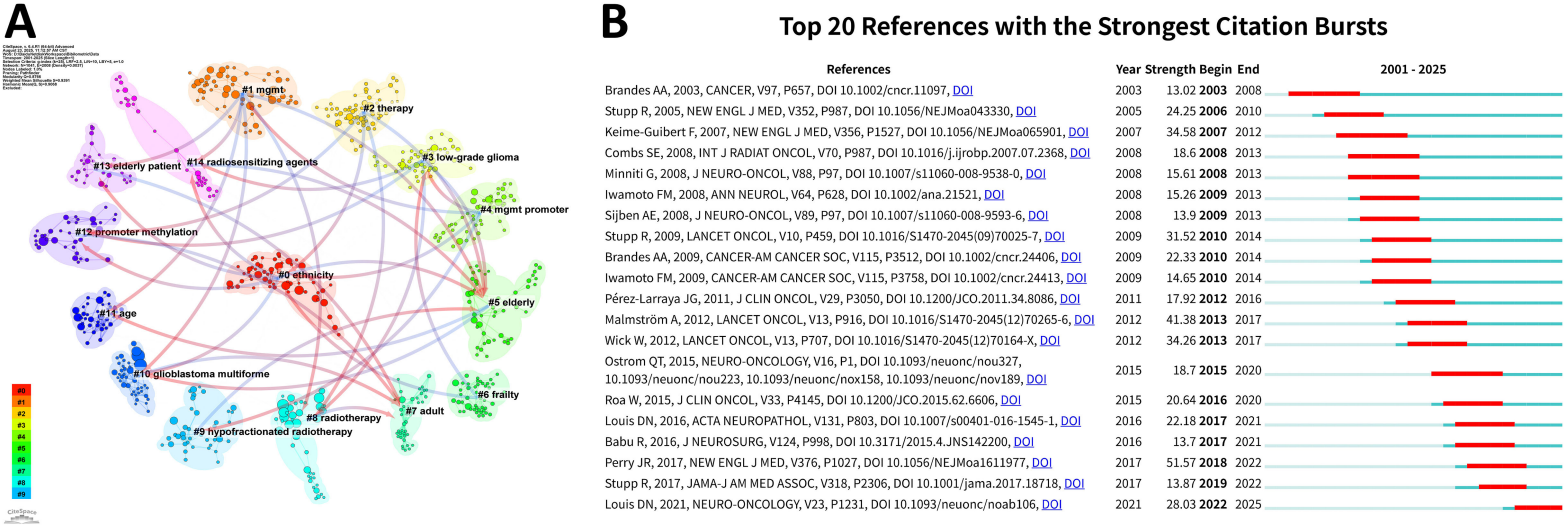
Co-citation network and burst analysis of references in elderly glioma research. **(A)** CiteSpace cluster visualization (time slice: 2001–2025, one year per slice; selection criteria: top 50 most-cited references per slice; pruning: pathfinder). Clusters labeled by log-likelihood ratio (LLR) algorithm with manual verification against citing article content. Modularity Q = 0.8766 (Q>0.7 indicates significant cluster structure); weighted mean silhouette S = 0.9391 (S>0.7 indicates homogenous clusters). **(B)** Top 20 references with strongest citation bursts detected by Kleinberg’s algorithm (minimum duration: 2 years; minimum burst strength: 3.0). Red bars indicate burst duration; strength values represent burst intensity.

Generally, we could identify three research phases: (1) Histopathological classification (2001–2013): This period corresponds to the initial focus on tumor classification and basic understanding of glioma biology in elderly patients. (2) Therapy standardization with radiotherapy/temozolomide (2014–2018): This phase is anchored by the publication of pivotal clinical trials including the NOA-08 trial (2012) (11) and Nordic trial (2012) (12), which established the role of temozolomide (TMZ) and radiotherapy (RT) in elderly glioma, and the CE.6 trial (2017) which further refined treatment approaches (13). (3) molecular stratification focusing on MGMT methylation (2019–2025): This phase is supported by the emergence of MGMT promoter methylation as a critical biomarker, evidenced by the citation burst analysis (**Figure 3B**) and the CeTeG/NOA-09 trial (2019) (14). These three research phases represent a descriptive interpretation of the field’s evolution, supported by key clinical trials and citation burst analysis, rather than a statistically derived classification.

These findings highlighted the increasing academic and clinical interest in elderly glioma, reflecting its growing prominence as a significant area of research and its potential therapeutic implications within the field of oncology. The three research phases represent a descriptive interpretation of the field’s evolution, supported by key clinical trials and citation burst analysis.

### 3.2 Country/region distribution

**Figure 4:**
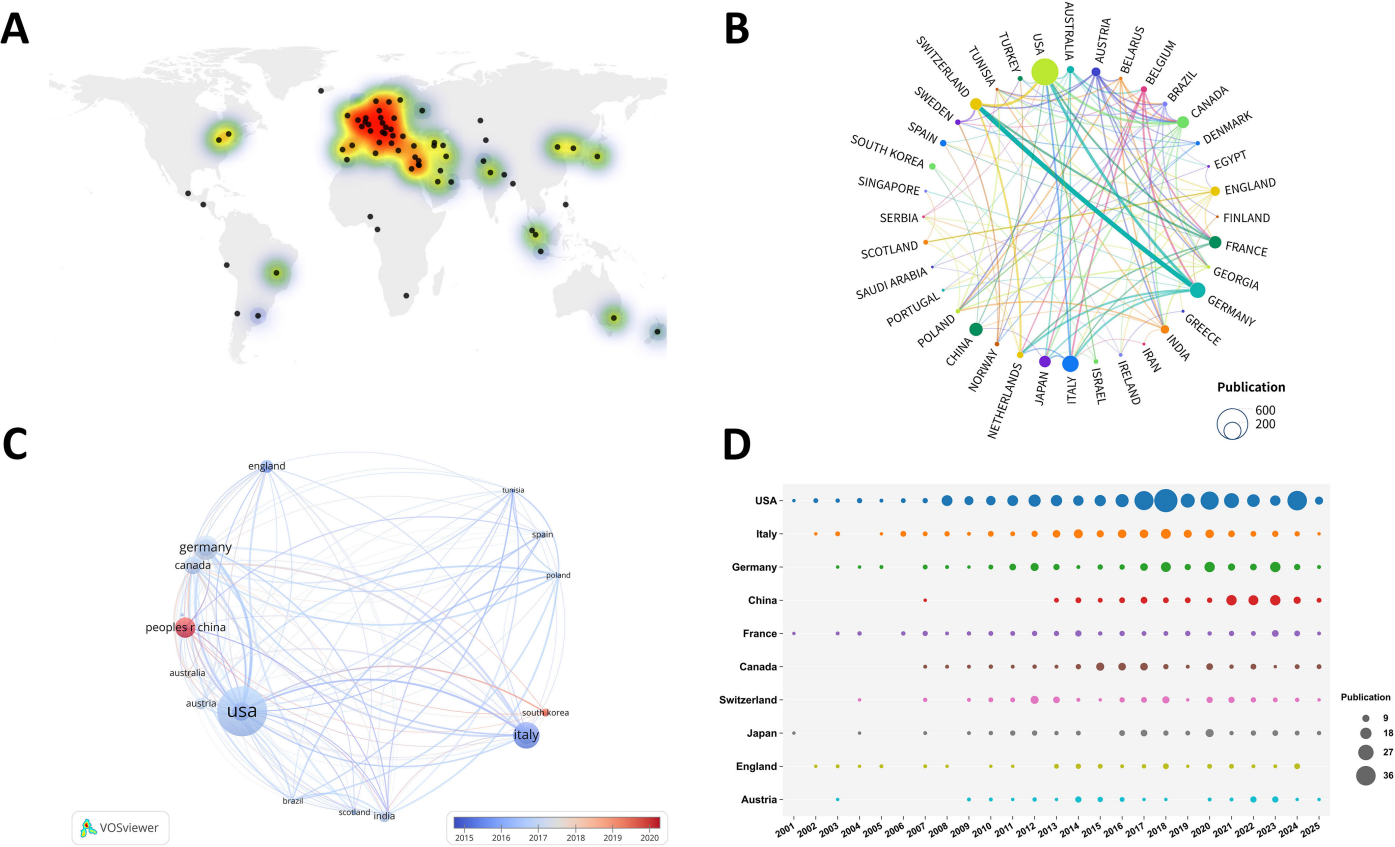
Global distribution and international collaboration patterns in elderly glioma research. **(A)** Heat map of publication output by country (n=78 countries total). Color intensity represents publication count normalized by maximum value; black dots indicate institutional locations. **(B)** Chord diagram of international collaborations. Line thickness represents total link strength (TLS) calculated by VOSviewer based on co-authorship frequency; minimum threshold: 5 collaborative publications. Node size is proportional to national publication output. **(C)** VOSviewer network visualization. Nodes represent countries, size proportional to publication count. **(D)** Temporal bubble chart of top 10 countries. Bubble area proportional to annual publication count; color coding consistent across panels.

Research on elderly gliomas was conducted across 78 countries and regions, indicating a multicentric distribution pattern (**Figure 4A**). The top ten countries in terms of publication output were primarily developed nations, with China (106 publications, 8.2%) being a notable exception. Among these, the USA demonstrated the highest level of research activity in this field, with a publication count of 426 (32.8%), significantly exceeding that of other countries and regions. European countries, including Italy (161 publications, 12.4%), Germany (143 publications, 11.0%), France (93 publications, 7.2%), exhibited a substantial concentration of research efforts in this area (**Supplementary Table 2**).

### 3.3 International cooperation

The evaluation and visualization of international collaborations among active countries and regions were conducted using a network analysis approach (**Figures 4B, C**). The analysis included only those countries and regions with a minimum of eight publications. In the resulting network map, the thickness of the connecting lines indicated the strength of collaboration, while the size of the nodes represented the volume of publication output. The network visualization highlighted strong collaborative relationships between the United States and several other nations, such as Germany, Canada, England, and Italy (**Figure 4C**). Germany and Switzerland showed a strong cooperative relationship. Importantly, the proportion of international collaborative research increased markedly since 2015, indicating a growing level of global engagement in this area (**Figure 4D**). Noteworthy was China’s position, despite its initial publication emerging only after 2007, followed by a rapid increase in output. This trend suggested that elderly gliomas receive heightened attention in nations experiencing rapid population aging.

### 3.4 Organization distribution

Research institutions dedicated to the study of elderly gliomas were both diverse and numerous, with a total of 2079 institutions having published at least one pertinent paper. The top 15 organizations collectively contributed 355 publications, accounting for 27.3% of the total output (**Supplementary Table 3**). Reflecting the international distribution, the University of Zurich led in publication output (total publications, TP = 52), followed by the Mayo Clinic (TP = 34), the University of Toronto (TP = 32), and the German Cancer Research Center (TP = 25). A notable concentration of publication activity was observed among these institutions (**Supplementary Figure 1**). Switzerland, the United States, Canada, and Germany were identified as the primary geographical hubs for research and treatment of elderly gliomas.

### 3.5 Journal distribution

The examination of subject areas via dual-map overlay of journals elucidated the dynamic progression of knowledge and the interdisciplinary linkages within this domain (**Supplementary Figure 2A**). Prior studies predominantly concentrated on molecular biology and genetics (cluster #8), along with clinical specialties (cluster #6). Recent publications have begun exploring interdisciplinary connections to materials science, with emerging but limited focus on tumor microenvironment and immune aspects. While immunology-related terms appeared infrequently in our burst analysis (strength <2.0), they represent nascent research directions rather than established frontiers.

We conducted a bibliometric analysis of journals frequently publishing on elderly glioma, identifying 318 such journals. *Neuro-Oncology* led with 157 publications (12.1%) and an impact factor of 13.4, followed by the *Journal of Neuro-Oncology* with 130 publications (10.0%) and an impact factor of 3.1. Other notable journals with over 40 publications included the *Journal of Clinical Oncology* (48 publications, 3.7%), *International Journal of Radiation Oncology Biology Physics* (46 publications, 3.5%) and *World Neurosurgery* (41 publications, 3.2%). These findings were visually represented in **Supplementary Figure 2**, **Supplementary Table 4**, with co-citation patterns highlighting the prominence of these journals.

The visualization of heatmaps depicting the relationships among journals and co-cited journals was conducted using VOSviewer (**Supplementary Figures 2D, E**). **Supplementary Figure 2D** demonstrated the active collaboration between the *Journal of Neuro-Oncology* and other journals, such as *Neuro-Oncology*, *Cancer*, and *Journal of Neurosurgery*. Additionally, it highlighted the co-citation relationships between *Neuro-Oncology* and *Journal of Neuro-Oncology*, as well as the active collaborations of *Journal of Clinical Oncology* with *Lancet Oncology* and *New England Journal of Medicine*.

### 3.6 Authors and co-cited authors analysis

From 2001 to 2025, a total of 7016 co-authors made their own contributions to the research of elderly glioma. For the authors with top ten publications, Weller, Michael held the highest number of publications (n=37), followed by Wick, Wolfgang (n=21) and Reifenberger, Guido (n=15) (**Supplementary Table 5**). Co-cited authors referred to those individuals who were concurrently referenced in two or more academic papers, thereby establishing interconnections within the scholarly network. On the other hand, Stupp, Roger emerged as the most frequently co-cited author, followed by Wick, Wolfgang (**Supplementary Figures 4A, B**).

For co-authorship analysis, Wick, Wolfgang and Weller, Michael displayed the strong connections, highlighting their close collaboration (**Supplementary Figure 4C**). Furthermore, **Supplementary Figure 4D** demonstrated a significant collaboration between Stupp, Roger, and Weller, Michael. It was important to highlight that the two most frequently co-cited authors also displayed strong collaborative relationships.

### 3.7 Citation and co-cited references

For the five most highly cited references, Ohgaki H. ranked first in terms of total citations (**Table 1**). This co-citation analysis highlighted pivotal moments in the development of treatment for elderly glioma, including the introduction of TMZ and the establishment of treatment standards (**Supplementary Table 6**).

**Table 1:** Top 5 references with highest citations in the research of elderly glioma.

Rank	Article title	Journal	First author	Citations	Normalized citations	Publication year	Core content of results
1	Genetic pathways to primary and secondary glioblastoma	American Journal of Pathology	Ohgaki	1055	14.87	2007	Primary glioblastomas primarily impact the elderly and are genetically marked by 10q loss of heterozygosity (70%), EGFR amplification (36%), p16(INK4a) deletion (31%), and PTEN mutations (25%).
2	Temozolomide versus standard 6-week radiotherapy versus hypofractionated radiotherapy in patients older than 60 years with glioblastoma: the NORDIC randomized, phase 3 trial	Lancet Oncology	Malmstrom	934	14.33	2012	Conventional radiotherapy has been linked to suboptimal outcomes, particularly in patients over the age of 70. Therefore, both temozolomide and hypofractionated radiotherapy should be regarded as standard treatment modalities for elderly patients diagnosed with glioblastoma.
3	Temozolomide chemotherapy alone versus radiotherapy alone for malignant astrocytoma in the elderly: the NOA-08 randomized, phase 3 trial	Lancet Oncology	Wick	864	13.26	2012	Temozolomide is as effective as radiotherapy for treating elderly patients with malignant astrocytoma. MGMT promoter methylation may serve as a helpful biomarker for treatment outcomes and decision-making.
4	The definition of primary and secondary glioblastoma	Clinical Cancer Research	Ohgaki	802	17.33	2013	This review outlines the key features of primary and secondary glioblastomas, focusing on epidemiology, clinical aspects, histopathology, genetics, and expression, with an emphasis on the biological impact of IDH1 mutations. IDH1 genetic alterations serve as a definitive and more reliable diagnostic marker for secondary glioblastomas compared to clinical criteria.
5	Short-course radiation plus temozolomide in elderly patients with glioblastoma	New England Journal of Medicine	Perry	796	33.63	2017	Adding temozolomide to short-course radiotherapy improved survival in elderly glioblastoma patients compared to radiotherapy alone.

In the co-cited reference clustering analysis, the cluster labels delineated the research landscape of this field from a distinct perspective. These clusters provided an overview of the elderly glioma population, such as aging-specific characteristics (#6 frailty, #7 adult, #5 elderly, #11 age), pathological subtypes (#3 low-grade glioma, #10 glioblastoma multiforme), reflecting the core research foci and objectives in this domain. The remaining clusters focused on various therapeutic techniques, including first-line clinical treatments (#8 radiotherapy, #9 hypofractionated radiotherapy, molecular therapy advancements (#1 MGMT, #4 MGMT promoter), and new treatment methods (#3 radiosensitizing agents) (**Figure 3A**).

**Figure 3B** visually depicted significant citation bursts of references identified from 2001 to 2025, highlighting the evolution and trends of these references. We determined the occurrence, importance, and unexpectedness of mentioned sources (minimum duration 2). Each slice represents a year, organized by the burst’s start year, with strength-value indicating citation burst strength. The references citation burst analysis corroborated that therapeutic advance in elderly glioblastoma (GBM) evolved from foundation of chemo-radiotherapy to the establishment of approaches to novel interventions, such as updating molecular pathological category for CNS tumor and clinical use of bevacizumab.

This interdisciplinary nature of elderly glioma research fostered continuous knowledge exchange between different scientific communities, driving both conceptual innovation and practical breakthroughs in understanding and managing this complex condition. The integration of diverse scientific approaches contributed to a more comprehensive understanding of elderly gliomas while opening new avenues for therapeutic development.

### 3.8 Keywords

In the exploration of research domains, keywords encapsulated the central themes of an article. Analyzing these keywords unveiled research hotspots and directional trends. **Supplementary Table 7** presented the top 20 keywords based on co-occurrence frequency. We performed a cluster analysis on keywords with more than 25 occurrences using VOSviewer (**Supplementary Figure 3A**). The network graphs predominantly categorized the keywords into five distinct groups. The size of each circle indicated the frequency of the keyword’s appearance. From left to right, the first column represented the general characteristics of glioma, encompassing pathological types, geriatric features, survival, and related aspects. The second column emphasized glioma treatment, highlighting keywords such as radiotherapy, chemotherapy. The third column was characterized by terms commonly associated with molecular phenotypes, such as MGMT promoter methylation. The fourth column pertained to research on chemotherapy regimens, including TMZ and clinical trials. The fifth column pertained to treatment and prognosis, focusing on gross total resection.

**Supplementary Figure 3B** presented the identification of 25 significant keyword bursts from 2001 to 2025, visually depicting the evolution and trends of these keywords. We assessed the occurrence, significance, and novelty of the cited sources. Research hotspots that developed over time were identified, with early mentions of “astrocytoma”, “brain tumor” and “anaplastic astrocytoma”. Concurrently, the evolution of keywords highlighted landmark events across different periods, including pathological classifications, standardized treatment protocols, molecular marker analyses, and advancements in clinical trials.

To focus on the most significant and frequently used terms, a minimum occurrence threshold of 60 was implemented, reducing the initial set of 5,317 keywords. This refinement resulted in the identification of the 92 most prevalent keywords, which were subsequently categorized into five distinct clusters (**Supplementary Figure 3C**). This categorization aligned with the findings presented in **Supplementary Figures 3A**, where the density of keyword co-occurrence was illustrated based on frequency (**Supplementary Figure 3D**).

### 3.9 PubMed-indexed randomized controlled trials landscape

Between 2001 and 2025, a comprehensive search of PubMed identified 304 PubMed-indexed RCT records, thereby substantially enhancing the evidence base for glioma research. The management of glioma in elderly patients, with a particular focus on glioblastoma (GBM), has historically been challenged by the necessity to balance therapeutic efficacy with treatment tolerability. Over the past two decades, a succession of pivotal randomized controlled trials (RCTs) has progressively elucidated optimal therapeutic strategies. This has culminated in the development of an individualized, evidence-based approach that is informed by patient performance status and molecular markers, most notably the methylation status of the MGMT promoter.

Initial studies focused on establishing the role and optimal dosing of radiotherapy. Vuorinen et al. (15) demonstrated that resection conferred no significant survival benefit over biopsy alone in elderly patients with malignant glioma, supporting non-surgical management when safe resection is not feasible. Subsequently, Phillips (16) and Roa (17) independently compared conventional versus hypofractionated RT regimens and consistently showed that short-course RT (e.g., 40 Gy in 15 fractions or 35 Gy in 10 fractions)—a finding that became foundational for subsequent elderly-specific protocols.

With the established efficacy of TMZ in younger adults, its role in the elderly was investigated. Keime-Guibert et al. (18) conducted the first RCT in elderly GBM patients comparing RT alone, TMZ alone, and combined therapy, revealing comparable overall survival between TMZ monotherapy and RT (8.7 vs. 7.7 months), thereby positioning TMZ as a viable alternative to RT. This conclusion was reinforced by two landmark Nordic trials: Malmström (12) and Wick (11), which not only confirmed non-inferiority but also identified MGMT promoter methylation as a critical predictive biomarker: methylated tumors responded better to TMZ, whereas unmethylated tumors derived greater benefit from RT. This marked the advent of molecularly guided, personalized treatment in elderly GBM.

Building on these insights, the next question was whether RT and TMZ should be combined. Perry et al. (13) addressed this directly in the CE.6 trial, which randomized elderly GBM patients to short-course RT with or without concurrent and adjuvant TMZ. The combination significantly improved median overall survival (9.3 vs. 7.6 months), with benefit observed across MGMT subgroups—though more pronounced in methylated cases. This study established hypofractionated RT plus TMZ as the new standard for fit elderly patients eligible for combined modality therapy.

Concurrently, other approaches were explored. tumor-treating fields (TTFields) showed survival benefit in elderly subgroups of the EF-14 trial (19), offering an option for highly motivated, functionally robust patients. In contrast, trials adding bevacizumab to RT (20, 21) failed to improve overall survival, indicating it should not be used routinely in the first-line setting. Supportive care interventions, such as armodafinil for radiation-related fatigue, also yielded negative results (22, 23), highlighting ongoing challenges in symptom management.

**Figure 5:**
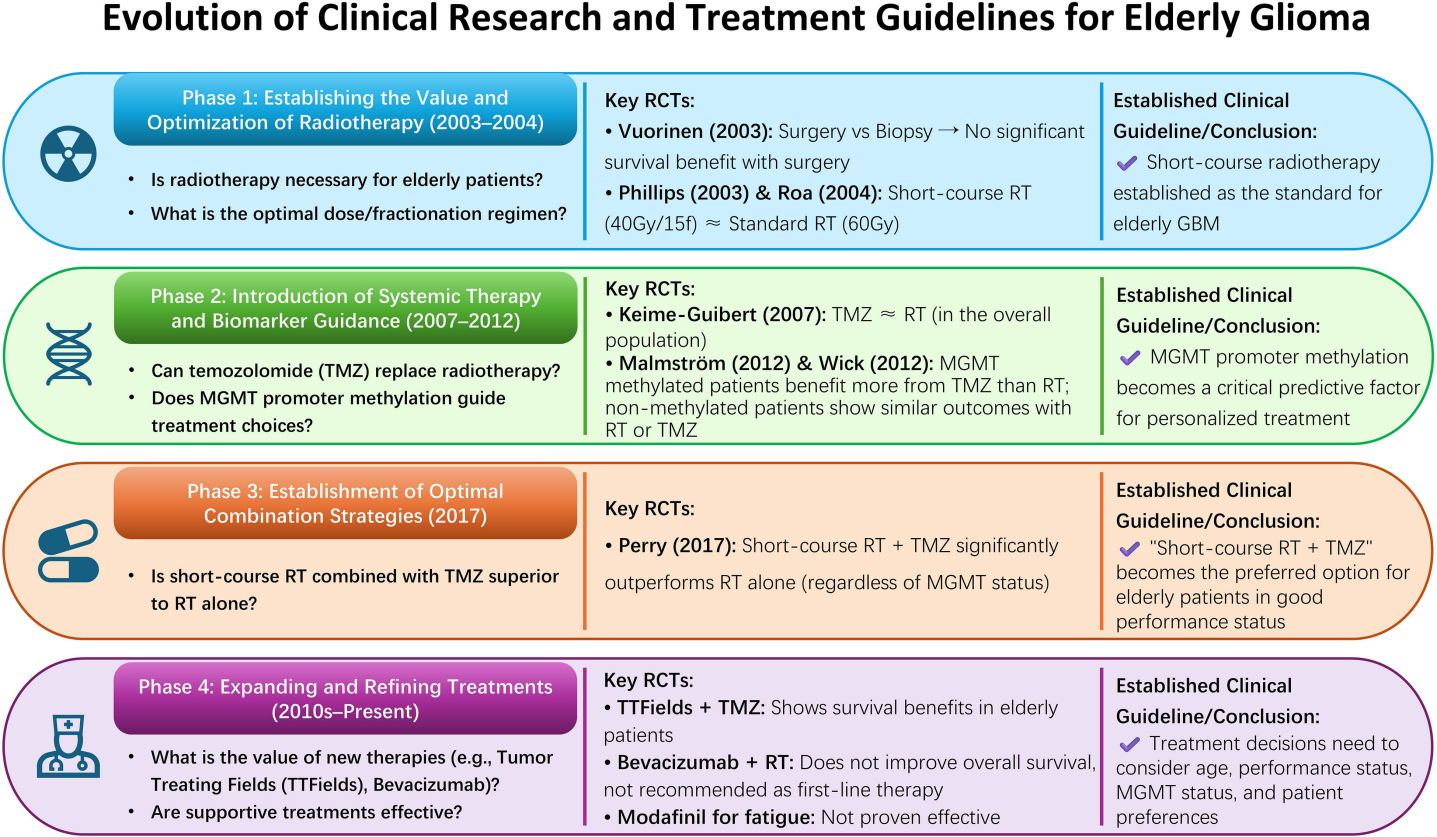
The evolution of clinical research and treatment guidelines for elderly glioma.

In summary, the current standard of care for elderly GBM evolved through a logical sequence of clinical questions: establishing the value and optimization of RT, validating TMZ as a non-inferior alternative and identifying MGMT as a key stratifier, demonstrating superior outcomes with combined hypofractionated RT and TMZ, and expanding the current treatment regimens (**Figure 5**). Together, these trials resolved several core clinical dilemmas—whether to treat, how to choose between modalities, and whether to combine them—culminating in a biomarker-driven, patient-centered treatment paradigm.

## 4 Discussion

This is the first study using bibliometrics to systematically analyze publications on elderly glioma over the past 25 years in WoSCC. This bibliometrics analysis provides a robust, comprehensive view of the burgeoning field of elderly glioma, offering both quantitative and qualitative insights.

With the accelerating global aging population, research on elderly glioma patients has progressively expanded in recent years. The fluctuating yet upward trend in research output since 2008 reflects growing academic and clinical attention to this special population. The first publication surge emerged in 2014, which directly correlated with pivotal clinical trials published during 2012–2013 investigating temozolomide chemotherapy regimens for elderly patients (24–26). The second research peak appeared in 2018, closely associated with molecular phenotype (MGMT) developments during this period. In subsequent years, publication volume remained consistently elevated in this field (14, 26).

Notably, both keyword bursts and research output bursts clustered around 2018, strongly indicating this period represented not merely quantitative growth but rather a qualitative transformation driven by paradigm shifts, methodological innovations, or significant advances (**Figure 3B**) (5, 27, 28).

Geographically, research activity showed multipolar characteristics. The publication distribution revealed active research clusters in Europe, North America and East Asia. USA had the highest publication output, while Swiss institutions showed the greatest centrality. European countries including Italy, Germany, France and the UK served as important research forces in this field. Notably, research from China has grown rapidly since 2013, reflecting both the country’s strengthening research capabilities and its direct connection to China’s rapidly aging population, which has driven expanding interest in elderly glioma research (28, 29).

The underrepresentation of low- and middle-income countries (LMICs) in geriatric glioma research underscores significant systemic inequities that compromise the generalizability and impact of global neuro-oncology studies. Key obstacles include disparities in research funding, inadequate scientific infrastructure, and limited access to high-impact journals and databases, which collectively impede the visibility and contributions of researchers from LMICs (30). To promote meaningful global participation, a comprehensive strategy is imperative. This strategy should encompass the establishment of open-access, multilingual data repositories to facilitate resource sharing, the development of funded international partnerships to strengthen local research capacity, and the encouragement of journals to implement equity-based policies, such as fee waivers and inclusive indexing.

In terms of international collaboration, academic cooperation between the United States and European countries appeared more substantial. China’s participation in international collaborative research has shown progressive growth in recent years (31). Research institutions demonstrated pronounced diverse characteristics. Bibliometric analysis clearly revealed the substantial contributions and leading position maintained by institutions such as the University of Zurich, Mayo Clinic, Duke University, Harvard Medical School (11, 32), characterized by well-connected and highly cited studies that outlined disciplinary progression.

The bibliometric analysis identified Weller, M. (n=37) and Wick, W. (n=21) as the most prolific authors, while Stupp, R. (n=919) and Wick, W. (n=404) ranked highest in co-citations—reflecting their foundational contributions to elderly glioma research (11, 33–35). Co-authorship networks involving internationally distributed experts facilitated knowledge dissemination and strengthened global collaborations essential for advancing this challenging field.

Current journal distribution primarily focused on oncology, neuroscience, surgery, and radiotherapy, with clinical oncology journals maintaining dominant influence. While our bibliometric analysis revealed limited current emphasis on immunological approaches in elderly glioma research, the appearance of related terms in recent publications suggests potential emerging interest that warrants monitoring in future analyses. For instance, newly developed technologies and nanomaterials in materials science may offer improved delivery systems for immunotherapy, while clinical applications of innovative therapies, like TTFields, presented promising opportunities for prognostic improvement in elderly patients (36, 37).

### 4.1 Hotspots and frontiers

Through cluster analysis of references and keywords, we can identify current research hotspots and potential future trends to help track academic developments and knowledge evolution. In elderly glioma research, the most frequently occurring keywords included “radiotherapy”, “temozolomide”, “elderly patients”, “glioblastoma”, “adjuvant chemotherapy”, and “quality of life” (3, 13, 26, 38–43). In recent years, new keywords have gradually emerged as research focuses, such as “phase II/III trials,” “promoter methylation,” and “tumor classification” (38, 44–48). These terms directly reflected advancements in molecular pathology and the continuous progress in clinical research.

Notably, clinical trials made significant contributions to the field of elderly glioma in recent years. Phase II and III studies, including radiotherapy-focused Phase II trials (49), concurrent chemoradiotherapy Phase III trials (13, 45), and investigations into agents like bevacizumab (50), collectively represented the progressive establishment of treatment paradigms for elderly glioma. Our citation burst analysis revealed that pivotal studies from 2014 and 2018 greatly influenced the trajectory of research. For instance, the NOA-08 study (2014) defined the therapeutic roles of TMZ and radiotherapy alone in elderly astrocytoma patients (11). The CeTeG/NOA-09 trial (2019) found that for newly diagnosed glioblastoma patients with MGMT promoter methylation, combining lomustine with temozolomide and radiotherapy significantly increased median overall survival compared to standard temozolomide, but there was no difference in progression free survival and higher toxicity (14). Meanwhile, the Stupp trial series has become the benchmark for GBM treatment, establishing the clinical value of key therapeutic approaches, such as concurrent chemoradiotherapy and TMZ (36, 44, 51). While new agents like bevacizumab failed to demonstrate survival benefits, they have shown meaningful improvements in quality of life (20, 50), representing an important shift in treatment strategies for elderly glioma patients.

From the prevalence of elderly glioma to poor prognosis, research consistently focused on GBM pathology and therapeutic approaches (52, 53). Following the established efficacy of radiotherapy, TMZ was incorporated as a first-line chemotherapeutic option (54, 55), highlighting the therapeutic response and prognostic significance of specific molecular subtypes, particularly those with MGMT promoter methylation (7, 8, 56).

### 4.2 Comparative therapeutic efficacy

Our bibliometric analysis reveals divergent citation trajectories for treatment modalities between elderly and general glioma literature. While anti-angiogenic therapy (bevacizumab) shows sustained citation growth in general GBM research, elderly-specific publications demonstrate declining attention post-2018, coinciding with negative RCT results (50). Conversely, TTFields show accelerating citation bursts in elderly subgroups (19), suggesting field recognition of age-specific applicability. These differential patterns underscore that therapeutic innovations cannot be assumed to translate across age strata without dedicated evaluation.

We caution that bibliometric prominence indicates research attention, not clinical efficacy. The absence of ‘immunotherapy’ or ‘checkpoint inhibitor’ keyword bursts in elderly glioma literature—despite explosive growth in general oncology—reflects genuine field constraint (poor CNS penetration, age-related immune senescence) rather than delayed adoption. Future innovation tracking should integrate bibliometric surveillance with clinical trial registry analysis (ClinicalTrials.gov) to distinguish publication activity from translational progress.

### 4.3 Perspectives for future research

Although research in elderly glioma has significantly advanced treatment paradigms in recent years, the deepening understanding of molecular mechanisms and pathology presents both new challenges and opportunities for future studies.

First, as knowledge of molecular subtypes and their prognostic significance grows, further exploration of the unique molecular phenotypes in elderly patients is essential. Emerging evidence suggests that elderly patients exhibit distinct molecular profiles and targetable features compared to other age groups, often leading to off-target effects or drug resistance in molecularly targeted therapies (57, 58). Second, the role of immune mechanisms in shaping treatment responses in this population warrants in-depth investigation. Key areas include the tumor immune microenvironment, mechanisms of drug resistance, immune evasion, and the potential of immunotherapies (59, 60). Third, technological advancements continue to drive the development of novel drugs and therapeutic approaches, offering new platforms for intervention. Innovations such as CAR-T cell therapy and TTF represent breakthroughs at both the cellular and physicochemical levels (36, 61–63). Although the limited presence of immunotherapy-related terms in our keyword burst analysis suggests that these approaches have not yet become established research foci in the study of glioma in the elderly, these advances represent potential future directions rather than current research priorities.

Currently, the integration of multi-omics data and AI-assisted analytical techniques holds promise for achieving transformative breakthroughs in elderly glioma treatment (6, 64, 65). AI is revolutionizing glioma management. Deep learning analyzes multimodal MRI for non-invasive IDH mutation prediction (66), the ROAM transformer model rapidly subtypes, grades, and genotypes billion-pixel histology slides (67), and intraoperative Raman-guided FastGlioma provides real-time tumor margin delineation (68). While our bibliometric analysis did not identify AI as an established research focus in elderly glioma literature, emerging applications in neuro-oncology suggest potential future directions. These perspectives represent forward-looking opinions rather than current bibliometric findings.

In addition, from clinical medicine and neurology perspectives, for the elderly population, psychological factors, social support systems, cognitive status and treatment compliance represented potential breakthrough research directions (10, 69, 70). Though not prominently represented in the bibliometric data, integration of geriatric assessment tools represents an important clinical consideration that merits increased research attention.

### 4.4 Limitations and interpretation

This bibliometric analysis is constrained by several methodological limitations. Firstly, there is a database and language bias: although we employed WoSCC and PubMed, which are among the most widely utilized sources, the exclusion of other databases such as Scopus and Embase may result in selection bias. Furthermore, our analysis was limited to English-language publications, thereby excluding non-English records and potentially overlooking significant research from non-Anglophone institutions. Secondly, there is an issue of keyword inconsistency and temporal bias: the variability in author-assigned keywords leads to fragmentation of conceptually related research, and manual normalization introduces a degree of subjectivity. Additionally, the reorganizations of the World Health Organization’s CNS tumor classifications in 2016 and 2021 create artificial discontinuities, complicating the attribution of phase transitions. Thirdly, bibliometric prominence does not equate to clinical validity. Bibliometric methodologies treat all indexed publications as equivalent units, irrespective of their empirical rigor. Our study includes both RCTs and observational studies. Citation metrics often correlate imperfectly with methodological quality, reflecting factors such as accessibility and journal impact factor rather than the strength of the evidence presented.

## 5 Conclusion​​

This study provides a comprehensive bibliometric analysis of current research trends and hotspots in elderly glioma. Recent years have witnessed a fluctuating yet overall upward trajectory in publication output, accompanied by substantially increasing citation rates. The research landscape demonstrates a multicentric distribution. Keywords analysis reveals that clinical treatments (radiotherapy and chemotherapy) and molecular markers (particularly MGMT status) constitute the predominant research foci in this field, with clinical studies assuming growing prominence. Future investigations may concentrate on molecular phenotypes, geriatric assessment tools, novel therapeutic approaches and geriatric-specific trial designs. Sustained, systematic exploration of this field will undoubtedly open new avenues for improving patient prognosis and therapeutic outcomes.

## Data Availability

The original contributions presented in the study are included in the article/**Supplementary Material**. Further inquiries can be directed to the corresponding authors.

## Supplementary material

Supplementary Figure 1 **(A)** Radar chart depicting the top nine institutions along with their countries. **(B)** Visualizing how institutions collaborate on elderly glioma research. Circle size reflects each institution’s publication output, and lines denote collaboration between them.

Supplementary Figure 2 **(A)** A dual-map overlay illustrates journals focused on elderly glioma. The Cleveland dot plot highlights the top 10 journals, ranked by publications **(B)** and citations **(C)**. The density map of journals **(D)** and co-cited journals **(E)** about elderly gliomas. **(F)** Top 20 cited journals with the strongest citation bursts.

Supplementary Figure 3 **(A)** In VOSviewer, keywords are depicted as nodes, with different colored vertical lines showing keyword clusters, and links between nodes representing keyword co-occurrence. **(B)** Top 20 keywords with the strongest citation bursts. **(C)** Thematic network maps depict key word trends related to elderly glioma research: **(D)** The visualization shows the density of keyword co-occurrence, with colors indicating different keyword clusters. The most frequent keywords are highlighted in yellow.

Supplementary Figure 4 Visual map of the author collaboration network. **(A)** Collaboration graph of authors who have published more than 5 papers. **(B)** This graph illustrates the collaboration network of 168 authors cited more than 20 times. The size of nodes and fonts is proportional to the number of articles published, and the line thickness shows the strength of collaboration between authors. **(C)** Jade block chart displaying the top 10 authors with the most publications. **(D)** A bubble chart showing the annual publication count for the top 10 authors.
