# Supplementary figures and images for: Mapping the global research landscape and innovations on elderly glioma: a bibliometric analysis
Source: Front Oncol. 2026 Mar 27;16:1769459. doi: 10.3389/fonc.2026.1769459 (PMC13065676; doi:10.3389/fonc.2026.1769459)

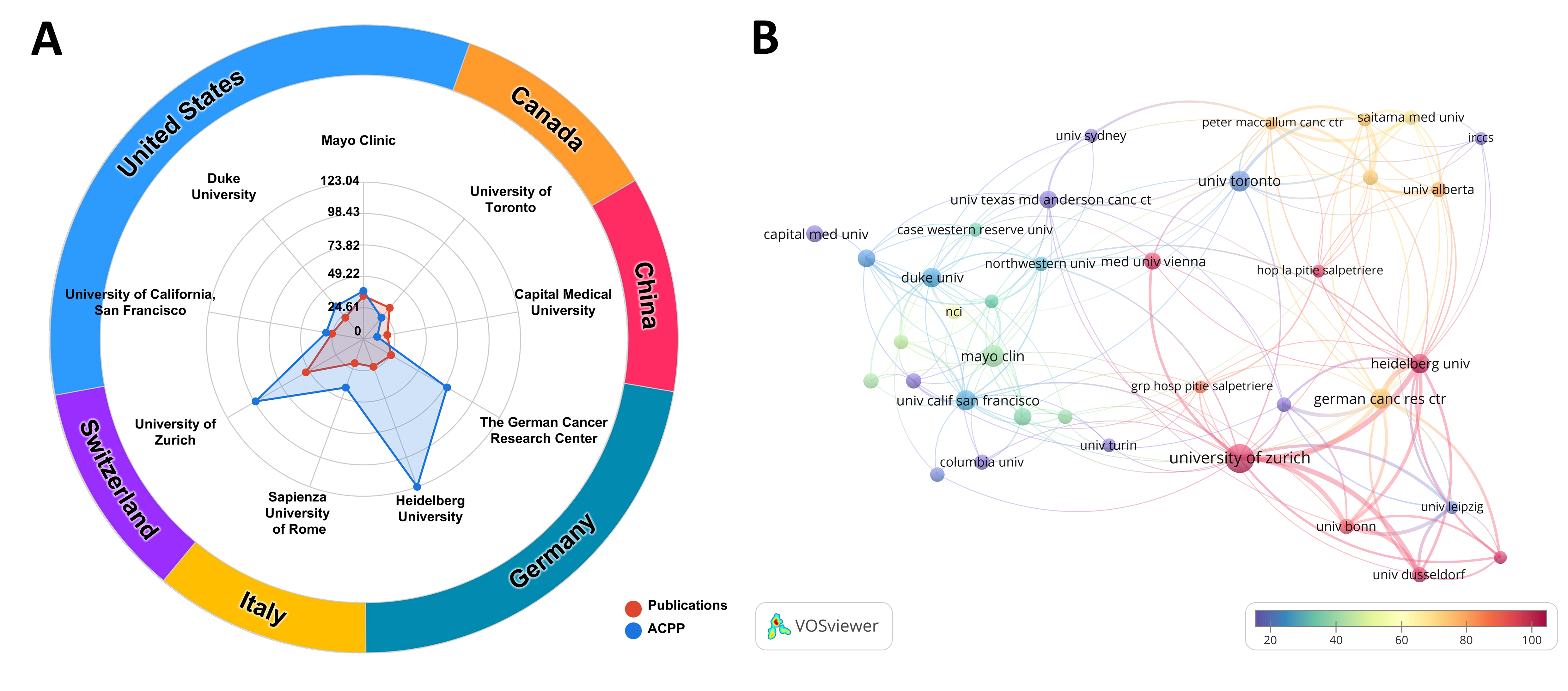

Supplement: Supplementary Figure 1 — (A) Radar chart depicting the top nine institutions along with their countries. (B) Visualizing how institutions collaborate on elderly glioma research. Circle size reflects each institution’s publication output, and lines denote collaboration between them. [file Image1.jpeg]

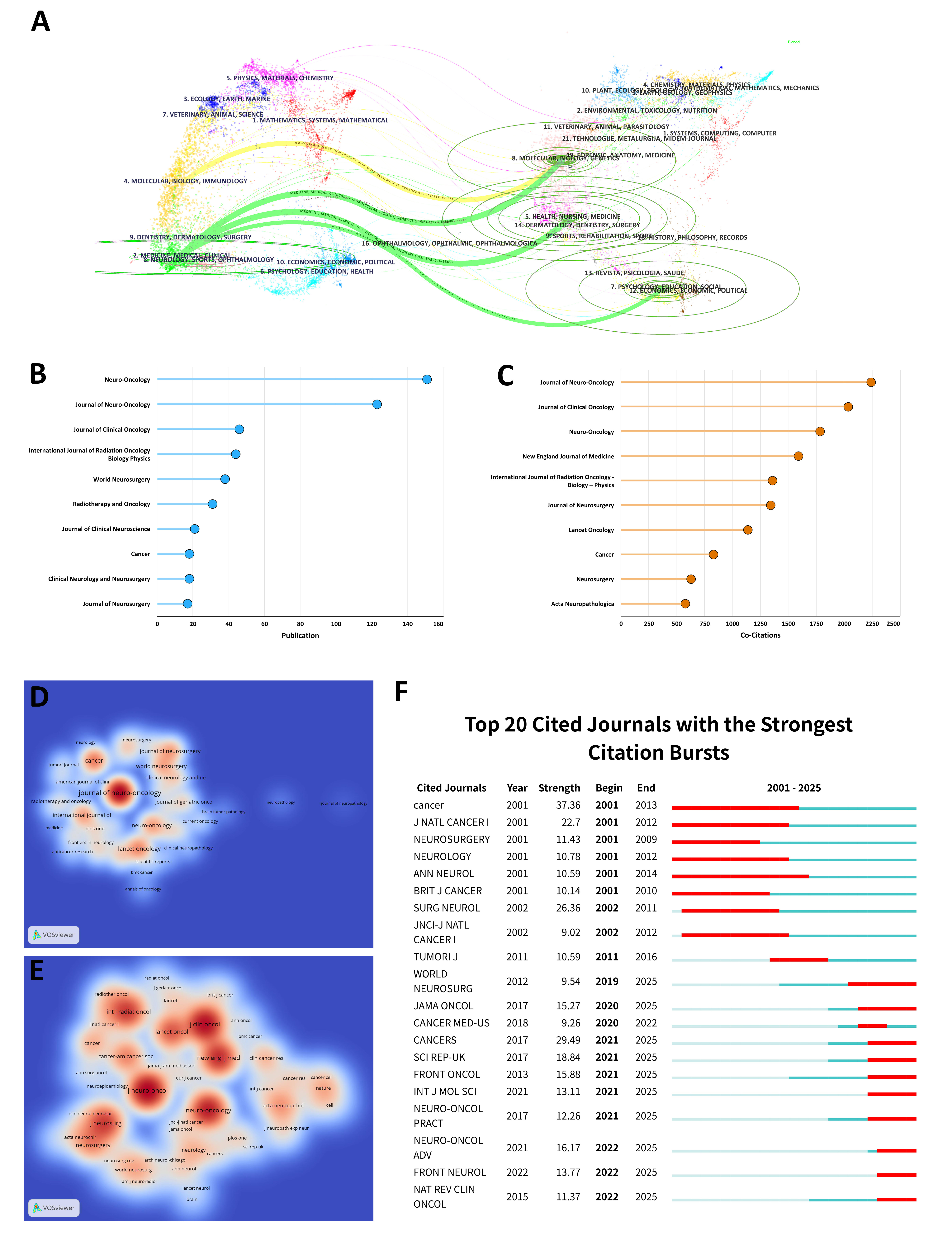

Supplement: Supplementary Figure 2 — (A) A dual-map overlay illustrates journals focused on elderly glioma. The Cleveland dot plot highlights the top 10 journals, ranked by publications (B) and citations (C). The density map of journals (D) and co-cited journals (E) about elderly gliomas. (F) Top 20 cited journals with the strongest citation bursts. [file Image2.jpeg]

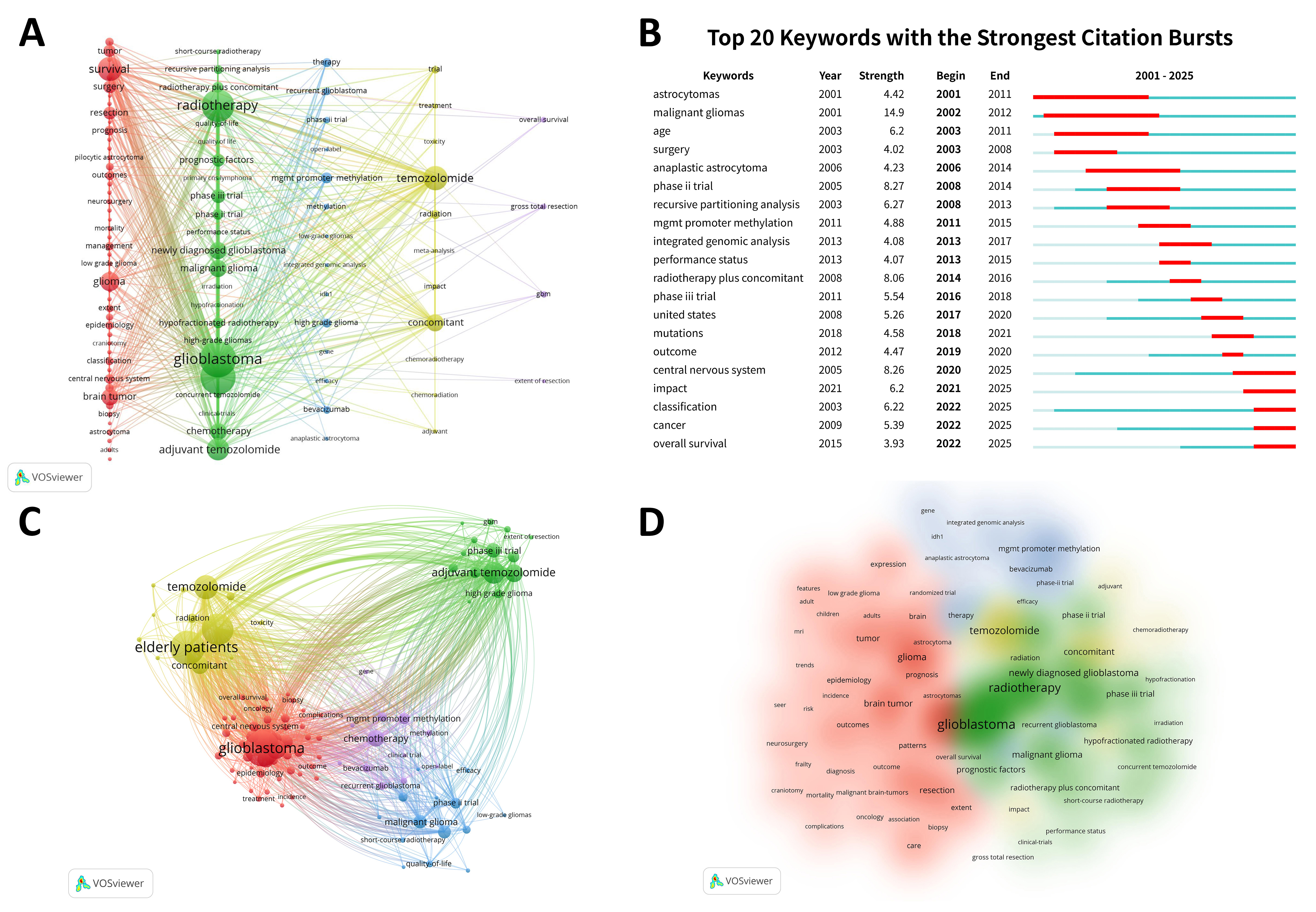

Supplement: Supplementary Figure 3 — (A) In VOSviewer, keywords are depicted as nodes, with different colored vertical lines showing keyword clusters, and links between nodes representing keyword co-occurrence. (B) Top 20 keywords with the strongest citation bursts. (C) Thematic network maps depict key word trends related to elderly glioma research: (D) The visualization shows the density of keyword co-occurrence, with colors indicating different keyword clusters. The most frequent keywords are highlighted in yellow. [file Image3.jpeg]

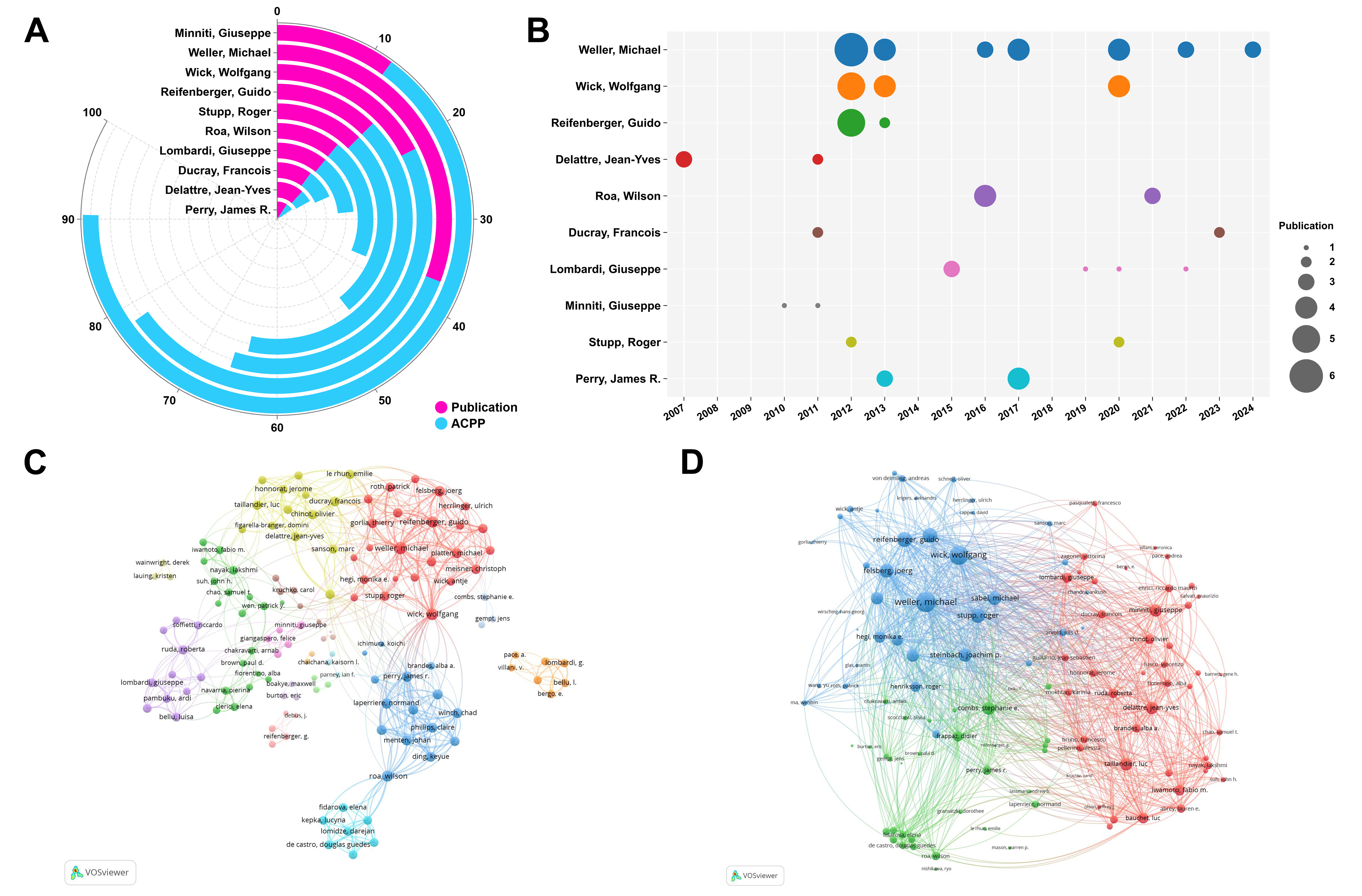

Supplement: Supplementary Figure 4 — Visual map of the author collaboration network. (A) Collaboration graph of authors who have published more than 5 papers. (B) This graph illustrates the collaboration network of 168 authors cited more than 20 times. The size of nodes and fonts is proportional to the number of articles published, and the line thickness shows the strength of collaboration between authors. (C) Jade block chart displaying the top 10 authors with the most publications. (D) A bubble chart showing the annual publication count for the top 10 authors. [file Image4.jpeg]
