# Supplementary material for: Mapping the global research landscape and innovations on elderly glioma: a bibliometric analysis
Source: Front Oncol. 2026 Mar 27;16:1769459. doi: 10.3389/fonc.2026.1769459 (PMC13065676; doi:10.3389/fonc.2026.1769459)
Supplement: Supplementary file 6 [file Table1.docx]

**Supplementary Table 1.** **Search strategies and retrieval formula in WoScc**

| **Set** | **Formula** |
| --- | --- |
| #1 | TS=(glioma* OR glioblastoma* OR astrocytoma* OR oligodendroglioma*) |
| #2 | AND TS=(elder* OR "aged patient*" OR geriatric* OR "older adult*") |
| #3 | NOT TS=(animal OR mice OR rat) |
| #4 | NOT TS=(orbital* OR meningoma*) |
| #5 | AND PY=(2001-2015) |
| #6 | AND DT=(“Article” OR "Meeting Abstract" OR "Review Article" OR “Editorial Material” OR “Letter”) |
| #7 | AND LA=English |

TS: Topic Search

PY: Publication year

DT: Document type

LA: Language

**Search strategies and retrieval formula in PubMed**

( ("glioma"[MeSH Terms] OR "glioblastoma"[MeSH Terms] OR "astrocytoma"[MeSH Terms] OR "oligodendroglioma"[MeSH Terms]) OR (glioma*[tiab] OR glioblastoma*[tiab] OR astrocytoma*[tiab] OR oligodendroglioma*[tiab]) ) AND ( ("Aged"[MeSH Terms] OR "Geriatrics"[MeSH Terms]) OR (elder*[tiab] OR "aged patient*"[tiab] OR geriatric*[tiab] OR "older adult*"[tiab]) ) NOT ("animals"[MeSH Terms] NOT "humans"[MeSH Terms]) AND ("English"[Language]) AND (("2001/01/01"[Date - Publication] : "2025/06/30"[Date - Publication]))

**Supplementary Table 2. Top 15 countries ranked by number of publications on elderly glioma**

| **Rank** | **Country** | **Publication** | **Citations** | **ACPP** | **TLS** |
| --- | --- | --- | --- | --- | --- |
| 1 | USA | 426 | 10107 | 23.73 | 159 |
| 2 | Italy | 161 | 4509 | 28.01 | 92 |
| 3 | Germany | 143 | 5988 | 41.87 | 175 |
| 4 | China | 106 | 1361 | 12.84 | 13 |
| 5 | France | 93 | 8022 | 86.26 | 102 |
| 6 | Canada | 85 | 2762 | 32.49 | 159 |
| 7 | Switzerland | 83 | 6500 | 78.31 | 150 |
| 8 | Japan | 81 | 1577 | 19.47 | 53 |
| 9 | England | 54 | 2081 | 38.54 | 48 |
| 10 | Austria | 48 | 2408 | 50.17 | 132 |
| 11 | India | 44 | 876 | 19.91 | 62 |
| 12 | Australia | 33 | 1099 | 33.30 | 56 |
| 13 | Netherlands | 27 | 2996 | 110.96 | 82 |
| 14 | South Korea | 26 | 239 | 9.19 | 13 |
| 15 | Spain | 26 | 988 | 38.00 | 13 |

ACPP: average citations per paper

TLS: total link strength

**Supplementary Table 3. The top 15 institutions ranked by number of publications on elderly glioma**

| **Rank** | **Institutions** | **Publications** | **Total Citations** | **ACPP** | **Country** |
| --- | --- | --- | --- | --- | --- |
| 1 | University Of Zurich | 52 | 5068 | 97.46 | Switzerland |
| 2 | Mayo Clinic | 34 | 1285 | 37.79 | United States |
| 3 | University Of Toronto | 32 | 702 | 21.94 | Canada |
| 4 | The German Cancer Research Center | 25 | 1892 | 75.68 | Germany |
| 5 | University Of California, San Francisco | 25 | 742 | 29.68 | United States |
| 6 | Heidelberg University | 23 | 2830 | 123.04 | Germany |
| 7 | Duke University | 22 | 738 | 33.55 | United States |
| 8 | Sapienza University of Rome | 20 | 806 | 40.30 | Italy |
| 9 | Capital Medical University | 19 | 208 | 10.95 | China |
| 10 | Cleveland Clinic | 19 | 537 | 28.26 | United States |
| 11 | University of Texas MD Anderson Cancer Center | 19 | 404 | 21.26 | United States |
| 12 | Medical University of Vienna | 18 | 1818 | 101.00 | Austria |
| 13 | Memorial Sloan-Kettering Cancer Center | 17 | 717 | 42.18 | United States |
| 14 | Columbia University | 15 | 215 | 14.33 | United States |
| 15 | University Calgary | 15 | 1055 | 70.33 | Canada |

ACPP: average citations per paper

**Supplementary Table 4. Top 10 Journals in terms of the number of published papers**

| **Rank** | **Journals** | **Publication** | **TC** | **ACPP** | **IF** | **Quartile in category** |
| --- | --- | --- | --- | --- | --- | --- |
| 1 | Neuro-Oncology | 157 | 1584 | 10.09 | 13.4 | Q1 |
| 2 | Journal of Neuro-Oncology | 130 | 4013 | 30.87 | 3.1 | Q2 |
| 3 | Journal of Clinical Oncology | 48 | 631 | 13.15 | 41.9 | Q1 |
| 4 | International Journal of Radiation Oncology Biology Physics | 46 | 574 | 12.48 | 6.5 | Q1 |
| 5 | World Neurosurgery | 41 | 483 | 11.78 | 2.1 | Q2 |
| 6 | Radiotherapy and Oncology | 31 | 220 | 7.10 | 5.3 | Q1 |
| 7 | Journal of Neurosurgery | 28 | 980 | 35.00 | 3.6 | Q1 |
| 8 | Journal of Clinical Neuroscience | 22 | 346 | 15.73 | 1.8 | Q3 |
| 9 | Neurology | 20 | 163 | 8.15 | 8.5 | Q1 |
| 10 | Cancer | 19 | 1670 | 87.89 | 5.1 | Q1 |

TC: total citation

ACPP: average citations per paper

IF: Impact factor

**Supplementary Table 5. Top 15 authors and co-cited authors in the research of elderly glioma**

| **Rank** | **Authors** | **Publication** | **TLS** | **TC** | **ACPP** | **Co-Cited Authors** | **Co-citations** | **TLS** |
| --- | --- | --- | --- | --- | --- | --- | --- | --- |
| 1 | Weller, Michael | 37 | 114 | 2922 | 78.97 | Stupp, R | 919 | 7205 |
| 2 | Wick, Wolfgang | 21 | 79 | 2348 | 111.81 | Wick, W | 404 | 4277 |
| 3 | Reifenberger, Guido | 15 | 71 | 1968 | 131.20 | Minniti, G | 382 | 3708 |
| 4 | Stupp, Roger | 15 | 35 | 1711 | 114.07 | Roa, W | 367 | 3491 |
| 5 | Delattre, Jean-Yves | 14 | 26 | 354 | 25.29 | Brandes, AA | 347 | 3119 |
| 6 | Lombardi, Giuseppe | 13 | 23 | 213 | 16.38 | Ostrom, QT | 329 | 2249 |
| 7 | Roa, Wilson | 13 | 23 | 379 | 29.15 | Malmström, A | 305 | 2909 |
| 8 | Ducray, Francois | 12 | 39 | 286 | 23.83 | Louis, DN | 277 | 1728 |
| 9 | Minniti, Giuseppe | 12 | 6 | 1211 | 100.92 | Perry, Jr | 267 | 2500 |
| 10 | Perry, James R. | 11 | 35 | 221 | 20.09 | Keime-Guibert, F | 266 | 2521 |
| 11 | Preusser, Matthias | 11 | 30 | 780 | 70.91 | Hegi, Me | 244 | 2450 |
| 12 | Ruda, Roberta | 11 | 24 | 133 | 12.09 | Weller, M | 242 | 2748 |
| 13 | Barnholtz-Sloan, Jill S. | 10 | 1 | 472 | 47.20 | Iwamoto, FM | 206 | 1885 |
| 14 | Felsberg, Joerg | 10 | 49 | 1223 | 122.30 | Chaichana, KL | 174 | 1603 |
| 15 | Iwamoto, Fabio M. | 10 | 8 | 531 | 53.10 | Chinot, Ol | 147 | 1641 |

TC: total citation

TLS: total link strength

**Supplementary Table 6. Top 5 references with highest co-citations in the research of elderly glioma**

| **Rank** | **First Author** | **Title** | **Journal** | **Year** | **TLS** | **Co-citation** | **Core contents or results** |
| --- | --- | --- | --- | --- | --- | --- | --- |
| 1 | Stupp R | Radiotherapy plus Concomitant and Adjuvant Temozolomide for Glioblastoma | New Engl J Med | 2005 | 2449 | 471 | Adding temozolomide to radiotherapy for newly diagnosed glioblastoma significantly improved survival with minimal extra toxicity. |
| 2 | Malmström A | Temozolomide versus standard 6-week radiotherapy versus hypofractionated radiotherapy in patients older than 60 years with glioblastoma: the Nordic randomised, phase 3 trial | Lancet Oncol | 2012 | 2009 | 303 | Standard radiotherapy led to poor outcomes, particularly in patients over 70. Temozolomide and hypofractionated radiotherapy should be standard treatments for elderly glioblastoma patients. |
| 3 | Wick W | Temozolomide chemotherapy alone versus radiotherapy alone for malignant astrocytoma in the elderly: the NOA-08 randomised, phase 3 trial | Lancet Oncol | 2012 | 1862 | 273 | Temozolomide is as effective as radiotherapy for treating elderly patients with malignant astrocytoma. MGMT promoter methylation may help predict treatment outcomes and assist in decision-making. |
| 4 | Roa W | Abbreviated Course of Radiation Therapy in Older Patients with Glioblastoma Multiforme: A Prospective Randomized Clinical Trial | J Clin Oncol | 2004 | 1945 | 266 | Survival rates are similar for patients receiving either standard or short-course RT, making the shorter RT a viable option for older GBM patients. |
| 5 | Keime-Guibert F | Radiotherapy for Glioblastoma in the Elderly | New Engl J Med | 2007 | 1910 | 261 | Radiotherapy modestly improves survival in elderly glioblastoma patients without affecting quality of life or cognition. |

TLS: total link strength

**Supplementary Table 7. Top 20 keywords in the research of elderly glioma**

| **Rank** | **Keywords** | **Occurrences** | **TLS** |
| --- | --- | --- | --- |
| 1 | Glioblastoma | 614 | 3804 |
| 2 | Elderly patients | 571 | 3708 |
| 3 | Radiotherapy | 512 | 3429 |
| 4 | Temozolomide | 322 | 2137 |
| 5 | Survival | 307 | 2056 |
| 6 | Adjuvant temozolomide | 262 | 1989 |
| 7 | Glioma | 193 | 988 |
| 8 | Newly diagnosed glioblastoma | 170 | 1338 |
| 9 | Concomitant | 166 | 1242 |
| 10 | Chemotherapy | 162 | 1181 |
| 11 | Phase III trial | 131 | 984 |
| 12 | Malignant glioma | 106 | 810 |
| 13 | Resection | 102 | 743 |
| 14 | Surgery | 101 | 663 |
| 15 | High grade glioma | 95 | 695 |
| 16 | Prognostic factors | 93 | 709 |
| 17 | Tumor | 90 | 433 |
| 18 | Brain tumor | 83 | 473 |
| 19 | MGMT promoter methylation | 80 | 588 |
| 20 | Radiotherapy plus concomitant | 79 | 632 |

TLS: total link strength
